# Integrative conjugative elements mediate the high prevalence of *tmexCD3-toprJ1b* in *Proteus* spp. of animal source

**DOI:** 10.1128/msystems.00429-23

**Published:** 2023-09-14

**Authors:** Kai Peng, Yangfan Li, Qiaojun Wang, Pengbin Yang, Zhiqiang Wang, Ruichao Li

**Affiliations:** 1 Jiangsu Co-Innovation Center for Prevention and Control of Important Animal Infectious Diseases and Zoonoses, College of Veterinary Medicine, Yangzhou University, Yangzhou, Jiangsu, China; 2 Joint International Research Laboratory of Agriculture and Agri-Product Safety, the Ministry of Education of China, Yangzhou University, Yangzhou, Jiangsu, China; 3 Institute of Comparative Medicine, Yangzhou University, Yangzhou, Jiangsu, China; California State University, Stanislaus, Turlock, California, USA

**Keywords:** SXT/R391 ICE, *Proteus*, *tmexCD-toprJ*, surveillance

## Abstract

Integrative conjugative elements (ICEs) are important mobile elements that are associated with the dissemination of antibiotic resistance genes (ARGs) in *Proteus*. Recent studies demonstrated that the tigecycline resistance gene cluster *tmexCD-toprJ* has emerged in ICEs of *Proteus*. However, the prevalence of *tmexCD-toprJ* positive *Proteus* from animal sources is unclear. To cover the gap, a total of 762 *Proteus* spp. were isolated from animal source from six provinces of China to identify the *tmexCD-toprJ* positive isolates. Eight *tmexCD-toprJ* positive isolates were identified, with the *tmexCD-toprJ* positive rate of 1.05% against all *Proteus* spp. and 2.79% against ICE-bearing *Proteus* spp. The *tmexCD-toprJ* gene cluster in these *Proteus* spp. were *tmexCD3-toprJ1b* and all of them were carried by ICEs. Genetic structure analysis showed that *tmexCD3-toprJ1b*-bearing ICEs were complicated and plastic, but the *tmexCD3-toprJ1b* was specifically integrated into variable region III (VRIII) of ICEs with the help of integrases. Furthermore, we found that the *umuC* gene, which is presented in VRIII of ICEs and other genetic structures of many other bacterial genomes, was a hotspot for the integration of *tmexCD-toprJ*-bearing potential mobile elements. In summary, our results suggested that *Proteus* were important reservoirs for *tmexCD-toprJ* due to the high prevalence of ICEs. Therefore, continuous surveillance of ICEs-associated ARGs in *Proteus* is necessary and significant for controlling their future transmission.

**IMPORTANCE:**

The emergence and spread of *tmexCD-toprJ* have greatly weakened the function of tigecycline. Although studies have demonstrated the significance of *Proteus* as carriers for *tmexCD-toprJ*, the epidemic mechanism and characteristics of *tmexCD-toprJ* in *Proteus* remain unclear. Herein, we deciphered that the *umuC* gene in VRIII of SXT/R391 ICEs was a hotspot for the integration of *tmexCD3-toprJ1b*-bearing mobile genetic elements by genomic analysis. The mobilization and dissemination of *tmexCD3-toprJ1b* in *Proteus* were mediated by highly prevalent ICEs. Furthermore, the co-occurrence of *tmexCD3-toprJ1b*-bearing ICEs with other chromosomally encoded multidrug resistance gene islands warned that the chromosomes of *Proteus* are significant reservoirs of ARGs. Overall, our results provide significant insights for the prevention and control of *tmexCD3-toprJ1b* in *Proteus*.

## INTRODUCTION

Integrative and conjugative elements (ICEs) are a type of chromosomally mediated mobile genetic elements that can be replicated with the host chromosome and transferred through conjugation (1). To date, many ICEs with various genetic structures have been identified in different species of bacteria. According to the integrase homology and backbone genetic features, majority of ICEs were assigned to 28 families (2). The SXT/R391 ICE is the largest and most well-studied ICE family, sharing a conservative integrase that mediates site-specific integration into the *prfC* gene on the host chromosome (2, 3). The first SXT ICE was identified in a sulfamethoxazole and trimethoprim resistant *Vibrio cholerae* isolate O139 (4). R391 ICE was initially thought to be IncJ plasmid, which was discovered in a clinical *Providencia rettgeri* isolate in 1972 (5). SXT and R391 ICEs have a similar genomic skeleton structure and a fixed chromosomal insertion and recombination site. Hence, the two types of elements are classified as the same family, SXT/R391 ICE (6).

Currently, SXT/R391 ICEs have been found in many different genera of bacteria with low GC content, including *Vibrio*, *Proteus*, *Shewanella,* and *Providencia*, from humans, animals, and environments (7 – 12). A conservative genetic context of roughly 47 kbp and many variable regions make up the backbone of SXT/R391 ICE. The conservative areas encode a total of 52 genes involved in activities such as integration, excision, and conjugative transfer (13). Five insertion hotspots (HS1, HS2, HS3, HS4, and HS5) and five variable regions (VRI, VRII, VRIII, VRIV, and VRV) in ICEs are typically reported to have varying DNA sequences (14). In addition to regulating bacterial motility, antibiotic resistance, and heavy metal resistance in the host, it has been discovered that the common SXT/R391 variable region genes also encode for a toxin-antitoxin system that prevents the loss of ICEs from the host (8). Therefore, as self-transmissible elements, ICEs played an important role in host adaptation and horizontal genetic structure exchanges in bacteria.

*Proteus* is an aggressive Gram-negative bacteria belonging to Enterobacteriaceae family that is widely found in intestinal tracts, skin of humans and animals, as well as in sewage, soil, waste, and decomposing organic matter (15). The genus of *Proteus* is known to be human opportunistic pathogens and naturally susceptible to aminoglycosides, fluoroquinolones, and sulfamethoxazole while being inherently resistant to polymyxins, tigecycline, and tetracycline (16). Recently, multidrug-resistant *Proteus* harboring ICEs have increasingly emerged (16 – 18), implying that ICEs played an important role in the dissemination of antibiotic resistance genes (ARGs) in *Proteus*. Moreover, it has been discovered that SXT/R391 ICEs carry an increasing number of clinically significant ARGs, including *bla*_CMY-2_ (9, 19, 20), *tet*(X) (21 – 24), *bla*_NDM_ (25, 26), *tmexCD-toprJ* (27 – 30), and *cfr* (23, 31) in *Proteus*. ICEs-bearing *Proteus* is becoming important reservoirs for critical ARGs.

TMexCD1-TOprJ1 is a plasmid-mediated resistance-nodulation-division (RND) family efflux pump conferring resistance to tigecycline, which is first identified in *Klebsiella pneumoniae* (32). Subsequently, a *tmexCD1-toprJ1* variant *tmexCD3-toprJ1b* was identified in *Proteus mirabilis* (33). In addition, studies provided that *tmexCD-toprJ* tended to integrate into the VRIII region of ICE in *Proteus* (30, 34). To investigate the prevalence of *tmexCD-toprJ* in ICEs of *Proteus*, we isolated 762 *Proteus* spp. from 729 animal source samples from six provinces of China in 2020 and 2022. A total of 281 SXT/R391 ICEs positive isolates were identified, and among them, eight *tmexCD-toprJ* positive *Proteus* spp. were characterized with genomic analysis to decipher the current molecular epidemiology of *tmexCD-toprJ* positive ICEs.

## RESULTS

### The prevalence and characteristics of ICEs in *Proteus* spp. from animal sources

**Fig 1:**
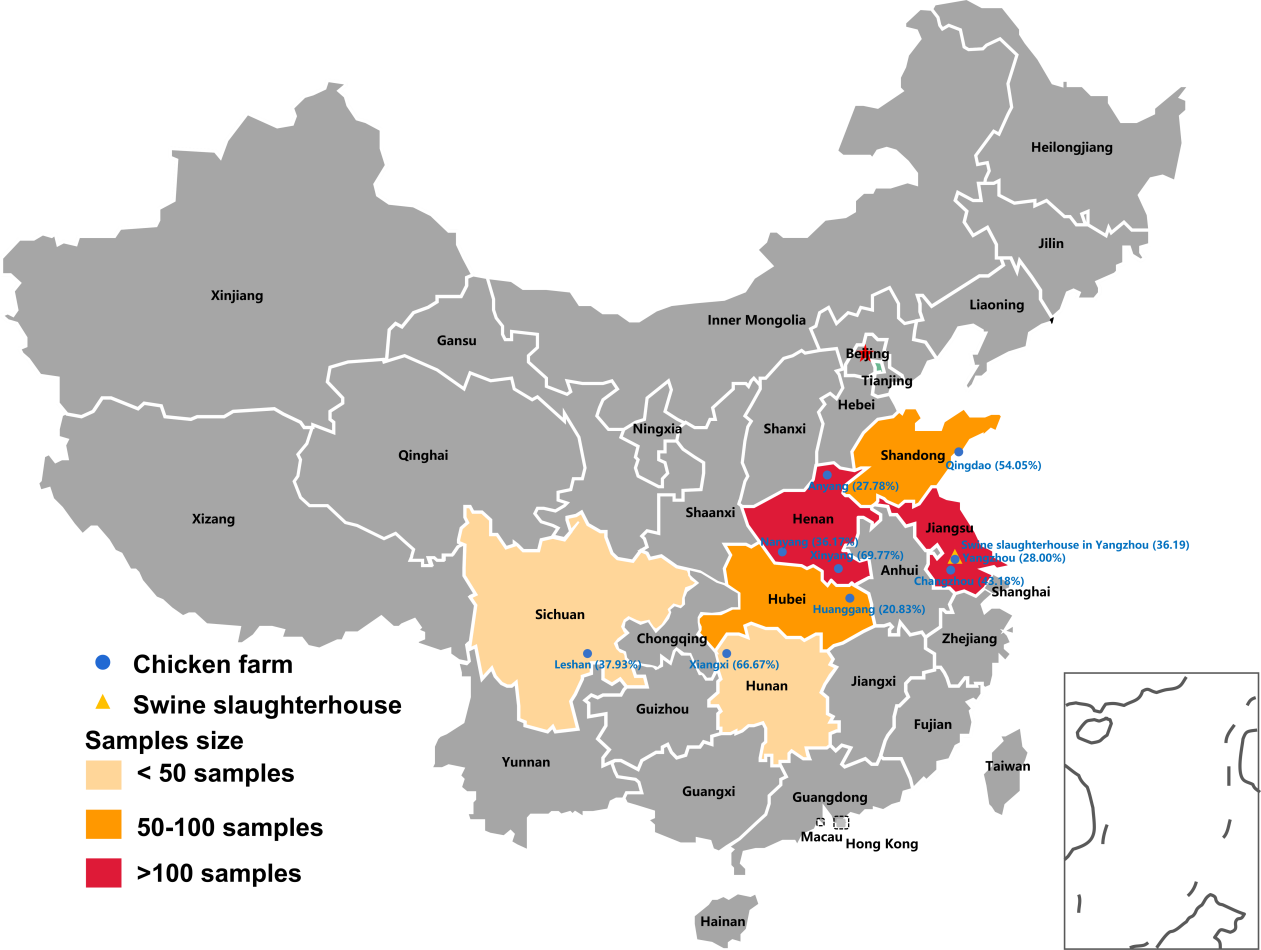
The distribution of collected samples from chicken farms and swine slaughterhouse in different provinces of China and the prevalence of ICEs in *Proteus* spp. of animal source. The fecal samples of chicken were collected from nine cities of six different provinces in China. The positive rate of ICEs in *Proteus* spp. of chicken farms and swine slaughterhouse from different cities were noted after the city’s name.

In 2020, 175 fecal and non-fecal samples (including blood, wastewater, and environmental samples) were collected from a swine slaughterhouse in Jiangsu Province. A total of 210 *Proteus* spp. were isolated from these samples according to the inherent resistance characteristics of tigecycline and colistin. Among them, 76 isolates were positive for SXT/R391 ICEs, and the positive rate was 36.19% (Fig. 1; Table S1). In 2022, a total of 552 *Proteus* spp. were isolated from 554 fecal samples collected in nine different chicken farms from six provinces of China, with 211 isolates testing positive for SXT/R391 ICEs. The prevalence of ICE-bearing *Proteus* spp. from different chicken farms varied from 20.83% to 69.77% (Fig. 1; Table S2). The overall positive rate of *Proteus* spp. harboring SXT/R391 ICEs from chicken farms was 38.22%. In general, the prevalence of ICEs in *Proteus* spp. from swine source and chicken source was similar and at high level. Species analysis showed that the 287 ICEs positive *Proteus* spp. consisted of four different species, including *Proteus mirabilis*, *Proteus vulgaris*, *Proteus terrae,* and *Proteus penneri*, with *Proteus mirabilis* dominated (89.20%, 256/287) (Fig. S1). Antimicrobial susceptibility testing showed that these ICEs positive *Proteus* spp. were 100% resistance to trimethoprim-sulfomethoxazole, tigecycline, amoxicillin, chloramphenicol, and colistin. The resistance rate of ICE-positive *Proteus* spp. was higher in chicken farms than that in the swine slaughterhouses (Fig. S1).

### The prevalence of *tmexCD-toprJ* in ICE-bearing *Proteus* spp. and the resistance phenotype of *tmexCD-toprJ* positive *Proteus* spp.

A total of eight *tmexCD-toprJ* positive isolates were identified from 762 *Proteus* spp., consisting of seven *P. mirabilis* isolates from chicken farms, and one *P. terrae* isolate from a swine slaughterhouse (Table 1). Among the seven *tmexCD-toprJ* positive isolates of chicken source, three were isolated from a chicken farm in Changzhou, Jiangsu. Another four were isolated from a chicken farm in Nanyang, Henan (Table S2). Of note, the eight isolates were all found in ICEs positive *Proteus* spp. The positive rate of *tmexCD-toprJ* against all *Proteus* spp. and ICE-bearing *Proteus* spp. were 1.05% (8/762) and 2.79% (8/287), respectively. Antimicrobial susceptibility testing showed that the eight isolates were multidrug resistant bacteria. All of them were resistant to florfenicol, streptomycin, tetracycline, tigecycline, trimethoprim/sulfamethoxazole, amoxicillin, and colistin but were susceptible to meropenem. In addition, conjugation assay showed that ARGs carried by the eight isolates could not be transferred to recipient strain C600 under laboratory conditions.

**TABLE 1:** Genome characteristics of the *tmexCD-toprJ*-positive isolates from this study and the NCBI database.^*a*^

Isolate	Collection date	Source	Species	Assembly method	Sequencing platform	Chromosome or plasmid size	NCBI accession no.	Plasmid type	Resistance genes	SXT/R391	ICE length (bp)
CZP17	2022	Chicken feces	*P. mirabilis*	Unicycler	MinION, Illumina	Chromosome 4,134,677 bp	CP110371	-	*sul1*, ***tmexCD3-toprJ1b*** **,** ***erm*** **(42),** ***floR*** **,** ***tet*** **(A),** ***tetR*** **,** ***aph(6)-Id*** **,** ***aph(3')-Via*** **,** ***aph(3'')-Ib*** **,** ***sul2*** **,** *tet*(J), *catA4*, *arr-3*, *catB3*, *bla*_OXA-1_, *aac(6')-Ib-D181Y*, *mph*(A), *bla*_CTX-M-14_, *fosA3*, *aac(3)-Iva*, *aph(4)-Ia*, *sul2*, *floR*, *aadA2*, *cmlA1*, *aadA1*, *sul3*, *aph(3')-Ia*, *dfrA12*	ICEPmiChnCZP17	126,140
CZP26	2022	Chicken feces	*P. mirabilis*	Unicycler	MinION, Illumina	Chromosome 4,053,008 bp	CP110373	-	*catA4*, *tet*(J), ***bla***_**HMS-1**_ **,** ***sul2*** **,** ***aph(3'')-Ib*** **,** ***aph(6)-Id*** **,** ***aph(3')-Via*** **,** ***floR*** **,** ***erm*** **(42),** ***tmexCD3-toprJ1b*** **,** *aph(3')-Ia*, *sul1*, *aadA1*, *dfrA1*, *lnu*(F)	ICEPmiChnCZP26	111,487
						pCZP26-1 2,683 bp	CP110374	Col3M_1	*qnrD1*	-	-
CZP44	2022	Chicken feces	*P. mirabilis*	Unicycler	MinION, Illumina	Chromosome 4,060,092 bp	CP110372	-	*catA4*, *tet*(J), ***bla***_**HMS-1**_ **,** ***tmexCD3-toprJ1b*** **,** *lnu*(F), *dfrA1*, *aadA1*, *sul1*, *aac(3)-Iva*, *aph(4)-Ia*, *sul2*, *floR*, *aac(6')-Ib-cr5*, *arr-3*, *bla*_DHA-1_, *mph*(E), *msr*(E), *qnrA1*, *aadA2*, *dfrA12*	ICEPmiChnCZP44	94,095
NYP73	2022	Chicken feces	*P. mirabilis*	Unicycler	MinION, Illumina	Chromosome 4,134,743 bp	CP110377	-	*catA4*, *tet*(J), ***aadA2*** **,** ***ere*** **(A),** ***dfrA32*** **,** ***aac(3)-Iva*** **,** ***aph(4)-Ia*** **,** ***sul2*** **,** ***floR*** **,** ***erm*** **(42),** ***tmexCD3-toprJ1b*** **,** *tet*(A), *sul1*, *dfrA10*	ICEPmiChnNYP73	77,839
NYP69	2022	Chicken feces	*P. mirabilis*	Unicycler	MinION, Illumina	Chromosome 4,130,619 bp	CP110376	-	*catA4*, *tet*(J), ***aadA2*** **,** ***ere*** **(A),** ***dfrA32*** **,** ***aac(3)-Iva*** **,** ***aph(4)-Ia*** **,** ***sul2*** **,** ***erm*** **(42),** ***tmexCD3-toprJ1b*** **,** *tet*(A), *floR*, *sul1*, *dfrA10*, *aadA2*	ICEPmiChnNYP69	78,554
NYP6	2022	Chicken feces	*P. mirabilis*	Unicycler	MinION, Illumina	Chromosome 4,149,995 bp	CP110375	-	*catA4*, *tet*(J), ***aadA2*** **,** ***ere*** **(A),** ***dfrA32*** **,** ***aac(3)-Iva*** **,** ***aph(4)-Ia*** **,** ***sul2*** **,** ***floR*** **,** ***erm*** **(42),** ***tmexCD3-toprJ1b*** **,** *mph*(E), *msr*(E), *aph(3')-Ia*, *aac(6')-Ib-D181Y*, *bla*_OXA-1_, *catB3*, *arr-3*, *sul1*, *dfrA10*, *floR*, *tet*(A), *fosA3*, *bla*_CTX-M-3_, *bla*_TEM-1_	ICEPmiChnNYP6	78,465
NYP68	2022	Chicken feces	*P. mirabilis*	SPAdes	Illumina	-	JAPDOJ000000000.1	-	*catA4*, *tmexCD3-toprJ1b*, *aac(3)-Iva*, *aph(4)-Ia*, *sul2*, *floR*, *tet*(A), *sul1*, *dfrA10*, *msr*(E), *mph*(E), *bla*_TEM-1_, *bla*_CTX-M-3_, *erm*(42), *aac(6')-Ib-D181Y*, *bla*_OXA-1_, *catB3*, *arr-3*, *fosA3*, *ere*(A), *dfrA32*, *aph(3')-Ia*, *aadA2*, *tet*(J)	-	-
TP22	2020	Swine feces	*P. terrae*	Unicycler	MinION, Illumina	Chromosome 4,138,893 bp	CP116621.1		*aadA1*, ***tmexCD3-toprJ1b*** **,** ***erm*** **(42),** ***floR*** **,** ***tet*** **(A),** ***tetR*** **,** ***ant(2'')-Ia*** **,** ***aph(3'')-Ib*** **,** ***aph(3')-Via*** **,** ***aph(6)-Id*** **,** ***sul2*** **,** *bla*_OXA-4_, *dfrA1*, *hugA*, *tet*(J), *tet*(H)	ICEPciChnTP22	126,287
						pTP22-1 2,683 bp	CP116622.1	Col3M_1	*qnrD1*	-	-
RGF134-1	2019	Swine feces	*P. mirabilis*	Unicycler	MinION, Illumina	Chromosome 4,095,724 bp	CP066833	-	*catA4*, ***tmexCD3-toprJ1b*** **,** *catA1*, *bla*_TEM-1_, *sul2*, *floR*, *aac(3)-Iva*, *aph(4)-Ia*, *aph(3')-Ia*, *aph(3'')-Ib*, *aph(6)-Id*, *aac(3)-Iid*, *arr-3*, *sul1*, *bla*_OXA-1_, *catB3*, *aac(6')-Ib-D181Y*, *bla*_CTX-M-65_,*fosA3*, *aadA5*, *dfrA17*, *aadA1*, *sat2­_fam*, *dfrA1*	ICEPmiChnRGF134-1	92,212
						pRGF134-1-4kb 4044 bp	CP066834.1	Col3M_1	-	-	-
						pRGF134-1-5kb 5947 bp	CP066835.1	Col3M_1	*qnrD1*	-	-
SDQ8C180-2T	2018	Chicken feces	*P. terrae*	Unicycler	MinION, Illumina	Chromosome 3,959,345 bp	CP073356.1	-	***tmexCD3-toprJ1b*** **,** ***tet*** **(X6),** *tet*(H), ***aph(3'')-Ib*** **,** ***aac(3)-Iva*** **,** ***aph(4)-Ia*** **,** ***aph(6)-Id*** **,** ***aph(3')-Ia*** **,** *aac(3)-Iva*, *hugA*, *dfrA1*, ***sul2***	ICEPciChn180	128,452
						pSDQ8C180-1 192,359 bp	CP073357.1	-	*tet*(B), *msr*(E), *mph*(E), *tet*(D), *aac(3)-Iid*, *aph(3')-Ia*, *sul1*, *bla*_DHA-1_, *aadA2*, *dfrA12*, *floR*, *sul2*, *aph(4)-Ia*, *aac(3)-Iva*, *aadA1*, *dfrA1*, *lnu*(F)	-	-
						pSDQ8C180-2 5,202 bp	CP073358.1	Col3M_1	-	-	-
						pSDQ8C180-3 3,220 bp	CP073359.1	Col3M_1	-	-	-
T1010	2020	Clinical patient	*P. mirabilis*	Unicycler	MinION, Illumina	Chromosome 4,001,374 bp	CP095765.1	-	*catA4*, *tet*(J), ***sul2*** **,** ***aph(3'')-Ib*** **,** *aac(3)-Iva*, ***aph(6)-Id*** **,** ***aph(6)-Id*** **,** ***aph(3')-Via*** **,** *aph(3')-Ia*, ***floR*** **,** ***tmexCD3-toprJ1*** **,** *fosA3*, *aac(6')-Ib-D181Y*, *bla*_OXA-1_, *catB3*, *arr-3*, *sul1*, *bla*_CTX-M-65_, *aadA1*, *sat2_fam*, *dfrA1*	ICEPmiChnT1010	104,344
FZP4280	2021	Clinical patient	*P. mirabilis*	SPAdes	Illumina	-	JAMOJR000000000.1	-	***tmexCD3-toprJ1b*** **,** *aadA*, *aph(3')-Via*, *strAB*, *bla*_CTX-M-65_, *catA*, *dfrA*, *erm*(42), *floR*, *fosA3*, *sul1*, *sul2*, *tetJ*	ICEPmiChn4280	
SDQ8C113RT	2018	Chicken feces	*P. terrae*	SPAdes	Illumina	-	JAGSNW000000000.1	-	***tmexCD3-toprJ1b*** **,** ***tet*** **(X6),** *tet*(H), *aac(6')-Ib-cr5*, *aac(3)-Iva*, *aph(3'')-Ib*, *aph(4)-Ia*, *aph(6)-Id*, *arr-3*, *aadA1*, *bla*_DHA-1_, *hugA*, *aac(6')-Ib-cr5*, *qnrD1*, *msr*(E), *mph*(E), *sul1*, *sul2*, *floR*, *dfrA1*, *lnu*(F)	-	-
SDQ8C176-1T	2018	Chicken feces	*P. terrae*	SPAdes	Illumina	-	JAGSHZ000000000.1	-	***tmexCD3-toprJ1b*** **,** ***tet*** **(X6),** *tet*(B), *tet*(D), *tet*(H), *aac(3)-Iid*, *aac(3)-IV*, *aadA2*, *ant(3'')-Ia*, *aph(3'')-Ib*, *aph(4)-Ia*, *aph(6)-Id*, *bla*_DHA-1_, *hugA*, *qnrD1*, *msr*(E), *mph*(E), *sul1*, *sul2*, *floR*, *catA3*, *dfrA12*, *dfrA1*, *lnu*(F)	-	-

^*a*^ ARGs carried by ICEs are highlighted with bold characters.

### The genomic characteristics of *tmexCD3-toptJ1* positive *Proteus* spp.

To investigate the genomic features of the eight *tmexCD-toprJ* positive *Proteus* spp., whole genome sequencing was performed. Of which, the complete closed genomes of seven isolates except NYP69 were generated using both long-read and short-read sequencing methods. The genome sizes of the eight isolates were ranged from 4,059,985 bp to 4,149,995 bp. Genomic analysis showed that *tmexCD-toprJ* gene cluster in the isolates were all *tmexCD3-toprJ1b* variants. Apart from *tmexCD3-toprJ1b*, many other ARGs, including four extended-spectrum beta-lactamase (ESBL) genes *bla*_CTX-M-14_, *bla*_HMS-1_, *bla*_CTX-M-3_ and *bla*_CTX-M-65_, were also detected in these isolates, implying that *Proteus* spp. is an important reservoir for ARGs (Table 1). However, only two Col3M-type *qnrD1*-bearing plasmids were found in the eight isolates, indicating that the ARGs in these isolates were almost located on the chromosome.

Seven *tmexCD3-toprJ1b*-bearing ICEs were found in the complete genomes of the seven *Proteus* spp. Although isolate NYP68 was only sequenced with short-read method, the backbone of *tmexCD3-toprJ1b*-bearing ICE was found in short-read assembly. According to the genetic alignments, *tmexCD3-toprJ1b*-bearing ICE in isolate NYP68 showed highly similar to ICE*Pmi*ChnNYP73 in isolate NYP73. Hence, we confirmed that the *tmexCD3-toprJ1b* gene cluster in the eight isolates was located on chromosomal ICEs. The complete ICEs in the seven isolates ranged in length from 75,940 bp (ICE*Pmi*ChnNYP73) to 123,747 bp (ICE*Pci*ChnTP22), with an average length of 104,721 bp. In addition, these ICEs contained two to nine ARGs, indicating that many ARGs in these isolates were located on other genetic structures of their chromosomes (Table 1).

Subsequently, we analyzed the genetic structures of other chromosomal ARGs of the seven *tmexCD3-toprJ1b* positive isolates. We found that majority of them were carried by genetic islands such as integrons and composite transposons (Fig. S2). The sizes of the seven genetic islands ranged from 11 kbp to 84 kbp. The largest genetic island found in the seven isolates was a multidrug resistance transposon in isolate CZP17. It contained 20 different ARGs, including ESBL gene *bla*_CTX-M-14_. Complex class one integrons were detected in three isolates from Nanyang chicken farm, and a more complex structure of class one integrons was found in isolate NYP6, highlighting the variable structures of complex class one integrons.

### Genetic structure analysis of *tmexCD3-toprJ1b*-bearing SXT/R391 ICEs

**Fig 2:**
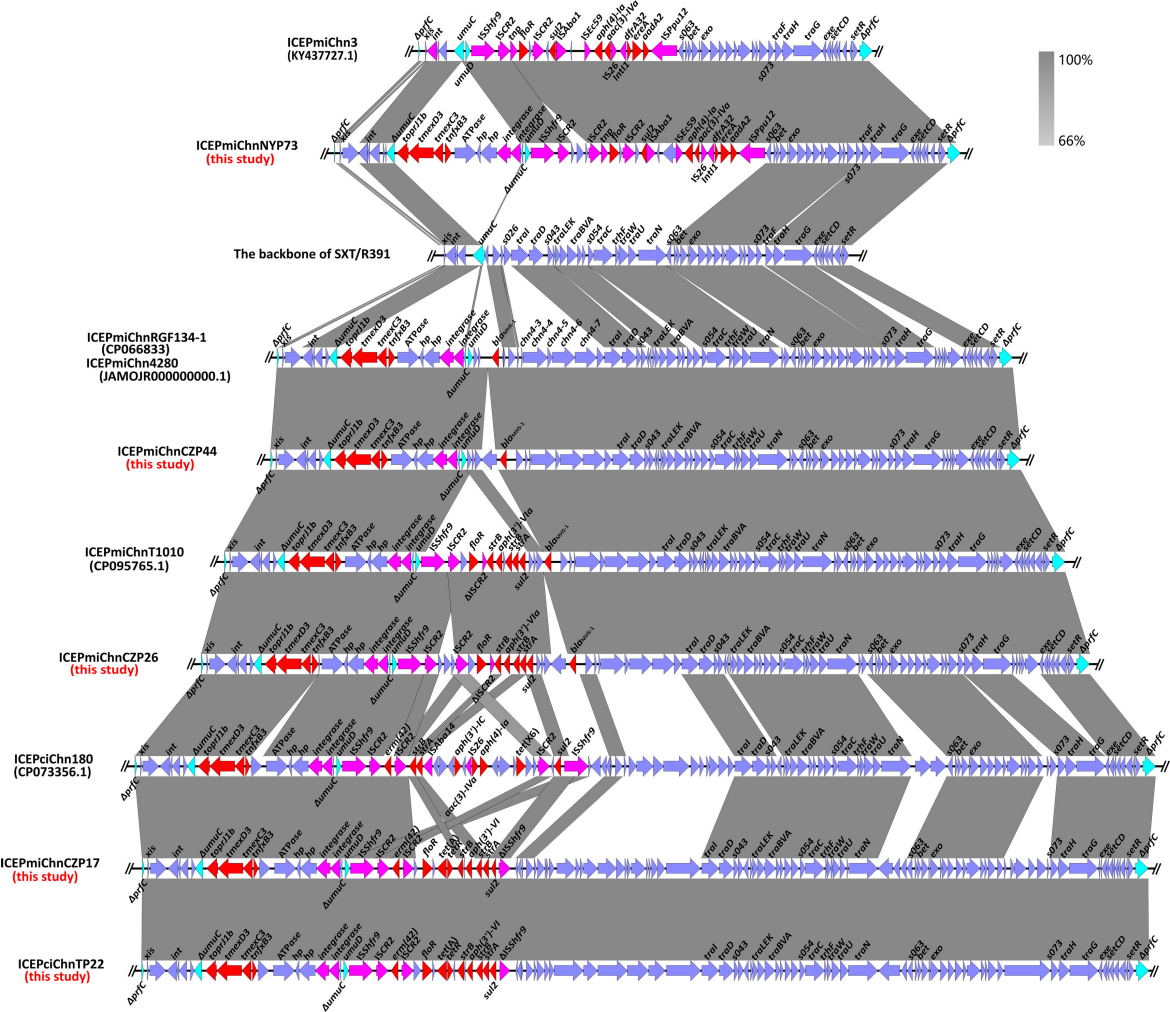
Genetic structures analysis of *tmexCD3-toprJ1b*-bearing SXT/R391 ICEs in this study and other similar ICEs. The gray-shaded region indicates the homologous region of the two ICE structures. The red arrows indicate the ARGs. The genes *pfrC*, *umuC,* and *umuD* are shown as cyan arrows. Pink arrows represent mobile elements. The gene *prfC* represents the insertion site of ICEs. The ∆ symbol indicates that the gene is truncated.

We identified seven *tmexCD3-toprJ1b*-bearing complete SXT/R391 ICEs in this study. Meanwhile, six previously reported *tmexCD3-toprJ1b*-bearing ICEs were retrieved from NCBI nr database and analyzed (Table 1). The comparative analysis found that ICE*Pmi*ChnNYP73, ICE*Pmi*ChnNYP69, and ICE*Pmi*ChnNYP6 detected in isolates from a chicken farm in Nanyang from this study share almost identical structures (Fig. S3). In addition, ICEs in isolates SDQ8C180-2T, SDQ8C176-1T and SDQ8C113RT from a chicken farm in Shandong also have almost identical gene arrangements (27). The phenomenon highlights that the *tmexCD3-toprJ1b*-bearing ICEs could widely spread in different niches via horizontally transfer or clonal spread. Notably, we found that *tmexCD3-toprJ1b*-bearing ICEs with different host species and geographical locations have a high degree of similarity, such that ICE*Pmi*ChnRGF134-1 and ICE*Pmi*Chn4280 with similar structures were isolated from a slaughterhouse and a patient, and ICE*Pmi*ChnCZP17 and ICE*Pmi*ChnTP22 with similar structures were carried by a *P. mirabilis* from chicken feces and a *P. terrae* from a slaughterhouse, respectively (Fig. 2).

Further analysis found that *tmexCD3-toprJ1b* gene cluster in these ICEs were all located on VRIII, and the core genetic environment of *tmexCD3-toprJ1b* was typical of a potential mobile structure *tmexCD3-toprJ1b-hp-hp-int-int* (Fig. 2; Fig. S4). The phenomenon also provided that the integration of *tmexCD3-toprJ1b* in VRIII of ICEs was likely assisted with integrase. Apart from *tmexCD3-toprJ1b*, many other ARGs were also located on VRIII. Due to the integration of foreign genes in insertion hotspots and variable regions, the sizes of these *tmexCD3-toprJ1b*-bearing ICEs were greater than the initial SXT/R391 ICEs identified in *V. cholerae* O139 and *P. rettgeri* R391 (Fig. 2). Hence, the SXT/R391 ICEs were mobile elements with consistent evolution ability. What is notable is that ICE*Pmi*ChnNYP73 had a significantly different backbone from other SXT/R391 ICEs. A total of 20 conserved genes involved in conjugation and other functions were lost in ICE*Pmi*ChnNYP73. Blastn analysis showed that ICE*Pmi*Chn3 was most similar to ICE*Pmi*ChnNYP73 (Fig. 2). Compared with ICE*Pmi*Chn3, a *tmexCD3-toprJ1b*-bearing region, with gene arrangement *tmexCD3-toprJ1b-hp-hp-int-int*, was integrated into the *umuC* gene of VRIII of ICE*Pmi*ChnNYP73. This further illuminated that the genetic structure of *tmexCD3-toprJ1b-hp-hp-int-int* was self-transmissible.

### Phylogenetic analysis of ICE-bearing *Proteus* spp.

**Fig 3:**
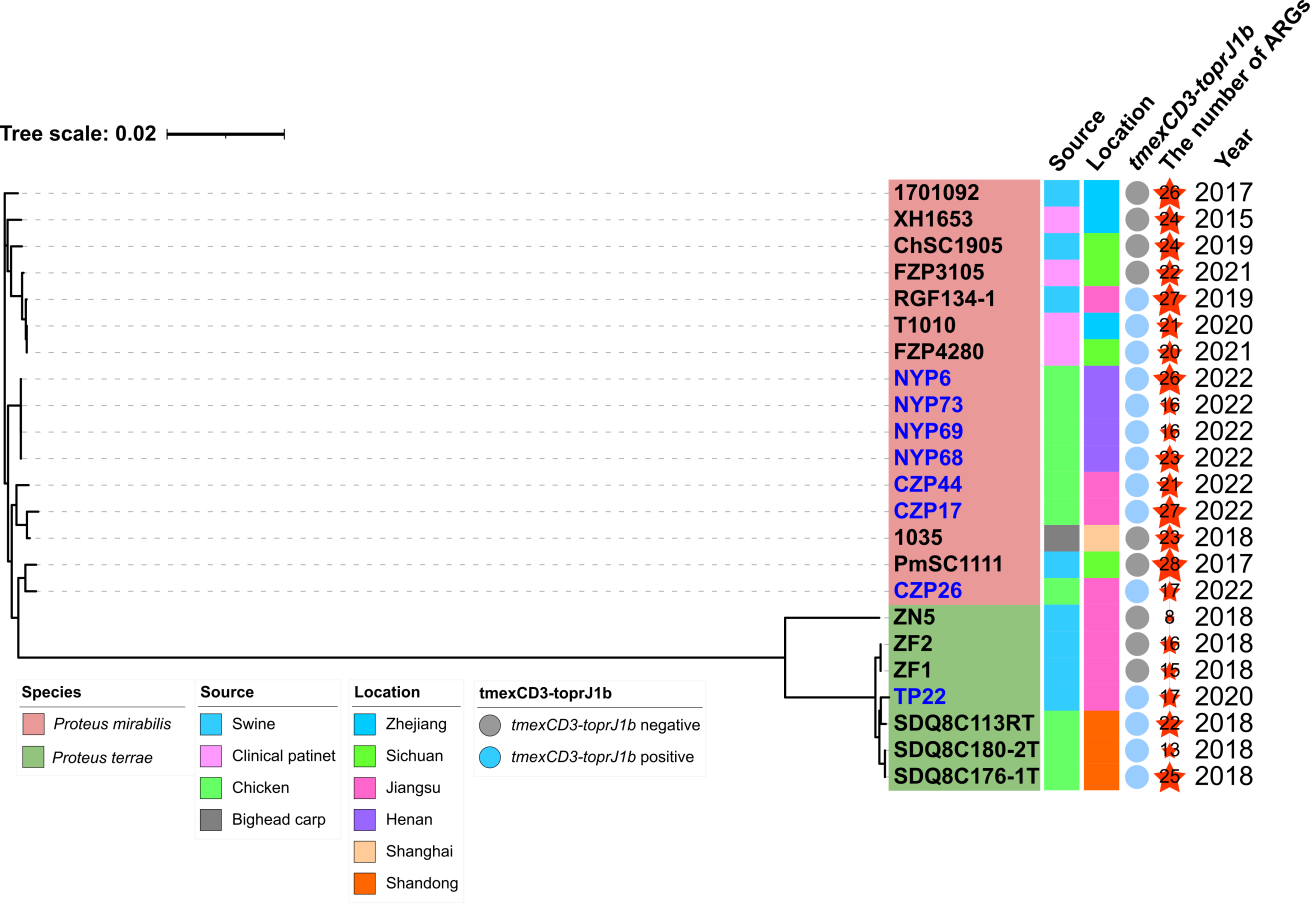
Phylogenetic analysis of *tmexCD3-toprJ1b* positive *Proteus* spp. and other ICE-harboring *Proteus* spp. The isolate names in this study were highlighted with blue. The ARGs numbers carried by isolates were represented by red stars of different sizes.

Many *tmexCD3-toprJ1b*-bearing ICEs have similar genetic structures. To investigate whether the structure of ICEs is related to the evolution of host *Proteus* spp., a phylogenetic tree consisting of all *tmexCD3-toprJ1b* positive *Proteus* spp. and some other *Proteus* spp. was constructed (Fig. 3). The four *P. mirabilis* isolates NYP6, NYP68, NYP69, and NTP73 from Nanyang chicken farm were clustered into one clade, implying that they originated from a common ancestor. Of note, the structures of ICEs in the four isolates were also almost identical, indicating that the spread of *tmexCD3-toprJ1b*-bearing ICEs in the farm was caused by the clonal transmission of these isolates. Isolates CZP44, CZP17, and CZP26 from a chicken farm in Changzhou were distributed in three different clades in phylogenetic tree and they carried different ICEs. This indicated that *tmexCD3-toprJ1b*-bearing ICEs in the farm were diversified. We found that some other ICE-harboring isolates were phylogenetically close to CZP17 and CZP26, showing that some *P. mirabilis* isolates were widely distributed in a variety of settings and served as important ICEs reservoirs. For isolate TP22, although it was closely related to isolates SDQ8C180-2T, SDQ8C113-RT, and SDQ8C176-1T in the phylogenetic tree, they harbored different ICEs. According to the results, the prevalence of ICEs in *Proteus* was complex and diverse. The *tmexCD3-toprJ1b* gene cluster was initially integrated into ICEs of different *Proteus* spp. and spread horizontally or vertically to different niches.

## DISCUSSIONS

In addition to the generally recognized plasmid-mediated transmission of most ARGs, SXT/R391 ICEs were also considered to be a significant factor in the spread of ARGs (35, 36). Our results showed that the prevalence of ICEs in *Proteus* from animal source is over 30%, which is higher than that in humans and food (10). Such highly prevalent ICEs in *Proteus* may accelerate the aggregation and horizontal transfer of ARGs within *Proteus*. Furthermore, many recent researches have demonstrated that *Proteus* were important hosts for tigecycline resistance gene cluster *tmexCD3-toprJ1b* (27, 29, 33). Meanwhile, *tmexCD3-toprJ1b* in *Proteus* is commonly carried by ICEs. Hence, ICEs were regarded as important carriers for the horizontal transfer of *tmexCD3-toprJ1b*. In this study, eight *tmexCD3-toprJ1b* positive isolates were identified from 762 *Proteus* spp., the positive rate was 1.05%, which was higher than that in clinical *Proteus* spp (29). Of note, the *tmexCD3-toprJ1b* gene cluster in the eight isolates was all located on ICEs. The positive rate of *tmexCD3-toprJ1b* in ICE-bearing isolates was 2.79%. It is amazing that nearly 3 out of every 100 ICE-bearing *Proteus* isolates of animal origin carried *tmexCD3-toprJ1b*. Our results warned that ICE-mediated *tmexCD3-toprJ1b* had high prevalence in *Proteus*. Apart from the surveillance of plasmid-mediated *tmexCD-toprJ* in other genera of bacteria (29, 37, 38), we also should take notice to monitor and prevent the prevalence of *tmexCD3-toprJ1b* in *Proteus*.

Source analysis of all available *tmexCD3-toprJ1b* positive *Proteus* isolates showed that the majority of them were isolated from animals. Only two isolates were isolated from humans. According to previous surveillance, the prevalence of *tmexCD3-toprJ1b* in clinical patients was at an extremely low level (0.2%, 1/437) (29). Comparatively, our investigation showed that the positive rate of *tmexCD3-toprJ1b* in *Proteus* isolates from animal sources was considerably higher than from human sources. The different prevalence of *tmexCD3-toprJ1b* in *Proteus* from human and animal sources is likely associated with the survival condition of bacteria. Of note, tigecycline was not allowed to be used in the animal industry. Whereas tetracycline was found in feed for animals in extremely high amounts (71,800 ± 8,860 µg/kg) (39), which could result in serious antibiotic residues in the intestinal or fecal environment of livestock and might lead to the selection and enrichment of ARGs, including *tmexCD3-toprJ1b*. We noticed that two human-source *tmexCD3-toprJ1b* positive isolates were closely related in the phylogenetic tree, and they were close to a porcine *tmexCD3-toprJ1b* positive isolate. More importantly, almost identical *tmexCD3-toprJ1b*-bearing ICEs were found in *Proteus* isolates from both animal and human sources. These phenomena implied that *tmexCD3-toprJ1b* positive isolates or *tmexCD3-toprJ1b*-bearing ICEs might have been transmitted between animals and humans. Hence, the rational use of antibiotics should be conducted in animals to curb the emergence of ARGs and prevent the transmission of ARGs of animal origin to humans.

Genetic structure analysis found that *tmexCD3-toprJ1b* has been integrated into many kinds of ICEs in *Proteus*. Meanwhile, we found that the core genetic environments of *tmexCD3-toprJ1b* in ICEs, *tmexCD3-toprJ1b-hp-hp-int-int*, were in line with the genetic environments of other *tmexCD-toprJ* variants in other genera of bacteria (37, 38, 40). The integration sites of these *tmexCD-toprJ*-bearing structures were mostly the *umuC* gene, suggesting that *umuC* was likely an integration hotspot for the two integrases. Meanwhile, the insertion site of VRIII of ICEs was also the gene *umuC*. This might explained that the high prevalence of *tmexCD3-toprJ1b* in ICEs. Although the phenomenon has been noticed in many studies, the transfer of *tmexCD3-toprJ1b-hp-hp-int-int* in different bacteria has not been proved with experiments.

We noticed that the majority of ARGs in the eight *tmexCD3-toprJ1b* positive *Proteus* spp. were located on chromosomes. However, only partly of them were carried by ICEs (Table 1). Hence, other ARGs-associated genetic structures in the chromosome of *Proteus* were also important to the dissemination of ARGs. Previous studies have demonstrated that many chromosomal genetic islands, including large transposons and some mobile genetic elements, were usually found in the chromosome of *Proteus*, and they could mediate the horizontal transfer of many ARGs (22, 41). In this study, we identified many different genetic islands harboring ARGs in the *tmexCD3-toprJ1b* positive *Proteus* spp., indicating that *Proteus* was important host for ARGs-bearing genetic islands. In addition, many *Proteus* were found to co-harbor chromosomal mobile elements and plasmids (23, 27). In consideration of the wide distribution of *Proteus* in natural environments, we should pay more attention to their role in the transmission and shelter of ARGs, especially significantly novel ARGs.

### Conclusion

In conclusion, a comprehensive investigation about the prevalence of *tmexCD-toprJ* in *Proteus* from animal sources was conducted. We discovered that the prevalent *tmexCD-toprJ* variant in *Proteus* was *tmexCD3-toprJ1b*. The high prevalence of *tmexCD3-toprJ1b* in *Proteus* was mediated by ICEs. The VRIII of ICEs was a hotspot for the integration of *tmexCD3-toprJ1b*-bearing genetic structures.

## MATERIALS AND METHODS

### Bacterial isolation

In 2020 and 2022, 729 samples were collected from a swine slaughterhouses and nine chicken farms from six provinces of China (Fig. 1; Table S1 and S2). The pretreatment of samples was performed as previously described methods (21). Briefly, the samples were incubated in 5 mL tryptic soy broth (TSB) for 6 hours. Then, *Proteus* was screened on tryptic soy agar (TSA) and MacConkey agar plates supplemented with 2 mg/L tigecycline and 4 mg/L colistin due to that they were inherently resistant to tigecycline and colistin. Suspected *Proteus* spp. were purified on TSA plats, and then confirmed by PCR targeting the genus-specific gene *tuf* and 16S rRNA gene with Sanger sequencing (42). The SXT/R391 ICE positive isolates were screened by PCR targeting conserved *int* genes (19). The tigecycline resistance gene cluster *tmexCD-toprJ* in *Proteus* spp. were screened by PCR using previously reported primers and positive PCR products were subjected to Sanger sequencing (28).

### Antimicrobial susceptibility testing and conjugation experiment

Antimicrobial susceptibility testing for commonly used antibiotics was performed by broth microdilution according to the Clinical and Laboratory Standards Institute guidelines (43). *Escherichia coli* ATCC25922 was used for quality control. To verify the transferability of *tmexCD-toprJ* in *Proteus*, conjugation assay was performed using rifampin resistant *E. coli* C600 (Rif^R^) as the recipient. The donor strain and the recipient strain in the log phase of growth were mixed at a ratio of 1:1 and then cultured overnight in 15-fold volume fresh LB broth or on LB agar plates. The transconjugants were screened on LB agar plates containing rifampin (300 mg/L), trimethoprim/sulfamethoxazole (4/76 mg/L), and tigecycline (2 mg/L). At last, the transconjugants were conﬁrmed by PCR for *tmexCD-toprJ* and 16S rRNA.

### DNA extraction, whole genome sequencing and bioinformatics analysis

Genomic DNA of the SXT/R391 ICEs positive isolates were extracted using the TIANamp Bacteria DNA Kit (Tiangen, China) following the manufacturer’s instructions. Then, we used Qubit 4 Fluorometer and NanoDrop (Thermo Scientific) to evaluate the quality and purity of genomic DNA, respectively. Following that, the genomic DNA was carried out short-read sequencing at Illumina Hiseq 2,500 platform and long-read sequencing at Oxford Nanopore Technologies MinION platform, respectively. Short-read genome assembly was performed with SPAdes (44). The closed complete genomes were obtained using a hybrid assembly strategy combining of short-read and long-read sequences with Unicycler (45). Plasmid replicon genes, insertion sequences and ARGs were investigated using abricate tool based on Plasmidfinder (46), ISfinder (47) and AMRFinder (48) databases. Subsequently, the genomes were annotated using Prokka and the resulting GFF3 files were pipelined into Roary to create core genome comparisons (49, 50). The phylogenetic trees of ICE-bearing *Proteus* spp. were constructed using Roary and FastTree based on SNPs of core genomes (50, 51). Easyﬁg was used to visualize the genetic comparisons (52).

## Data Availability

The genome sequences in this study were deposited into the National Center for Biotechnology Information, and the GenBank accession numbers are listed in Table 1.

## SUPPLEMENTAL MATERIAL

Supplemental material msystems.00429-23-s0001.docx Tables S1 to S3 and Figures S1 to S4.

ASM does not own the copyrights to Supplemental Material that may be linked to, or accessed through, an article. The authors have granted ASM a non-exclusive, world-wide license to publish the Supplemental Material files. Please contact the corresponding author directly for reuse.
