# Supplementary material for: Integrative conjugative elements mediate the high prevalence of tmexCD3-toprJ1b in Proteus spp. of animal source
Source: mSystems. 2023 Sep 14;8(5):e00429-23. doi: 10.1128/msystems.00429-23 (PMC10654056; doi:10.1128/msystems.00429-23)
Supplement: Supplemental material — Tables S1 to S3 and Figures S1 to S4. [file msystems.00429-23-s0001.docx]

**Supplementary data**

Table S1. The samples collected from Yangzhou slaughterhouse in Jiangsu Province in 2020 and the prevalence of *tmexCD3-toprJ1b* in these samples.

| Sources | Number of samples | Number of *Proteus* spp. | Number of SXT/R391 ICEs *Proteus* spp. | Positive rates^a^ | Number of SXT/R391 ICE *Proteus* spp. positive samples | Positive rates^b^ | Number of *tmexCD3-toprJ1* positive strains | Positive rates^c^ |
| --- | --- | --- | --- | --- | --- | --- | --- | --- |
| Non faeces | 9 | 8 | 0 | 0.00% | 0 | 0.00% | 0 | 0.00% |
| Faeces | 166 | 202 | 76 | 37.62% | 37 | 22.29% | 1 | 0.50% |
| Total | 175 | 210 | 76 | 36.19% | 37 | 21.14% | 1 | 0.48% |

**^a^** Ratio of SXT/R391 ICE *Proteus* spp. to total *Proteus* spp.

**^b^** Ratio of SXT/R391 ICE *Proteus* spp. to total samples.

**^c^** Ratio of *tmexCD3-toprJ1* positive isolates to total *Proteus* spp.

Table S2. The samples collected from chicken farms in different cities in China and the prevalence of *tmexCD3-toprJ1b* in these samples.

| Sources | Number of samples | Number of *Proteus* spp. | Number of SXT/R391 ICE *Proteus* spp. | Positive rates^a^ | Number of SXT/R391 ICE *Proteus* spp. positive samples | Positive rates^b^ | Number of *tmexCD3-toprJ1* positive isolates | Positive rates^c^ |
| --- | --- | --- | --- | --- | --- | --- | --- | --- |
| Anyang | 80 | 36 | 10 | 27.78% | 8 | 10.00% | 0 | 0.00% |
| Changzhou | 51 | 88 | 38 | 43.18% | 13 | 25.49% | 3 | 3.41% |
| Nanyang | 103 | 94 | 34 | 36.17% | 30 | 29.13% | 4 | 4.26% |
| Yangzhou | 100 | 125 | 35 | 28.00% | 30 | 30.00% | 0 | 0.00% |
| Qindao | 50 | 37 | 20 | 54.05% | 12 | 24.00% | 0 | 0.00% |
| Xinyang | 50 | 43 | 30 | 69.77% | 19 | 38.00% | 0 | 0.00% |
| Huanggang | 60 | 48 | 10 | 20.83% | 10 | 16.67% | 0 | 0.00% |
| Leshan | 30 | 58 | 22 | 37.93% | 14 | 46.67% | 0 | 0.00% |
| Xiangxi | 30 | 18 | 12 | 66.67% | 12 | 40.00% | 0 | 0.00% |
| Total | 554 | 552 | 211 | 38.22% | 148 | 26.71% | 7 | 1.27% |

**^a^** Ratio of SXT/R391 ICE *Proteus* spp. to total *Proteus* spp.

**^b^** Ratio of SXT/R391 ICE *Proteus* spp. to total samples.

**^c^** Ratio of *tmexCD3-toprJ1* positive isolates to total *Proteus* spp.

Table S3. Antibiotic susceptibility testing (mg/L) of eight *tmexCD3-toprJ1b* positive *Proteus* spp.

| IDs | Sources | Species | Antimicrobials(μg/ml) | | | | | | | | | | |
| --- | --- | --- | --- | --- | --- | --- | --- | --- | --- | --- | --- | --- | --- |
|  |  |  | AMX | MEM | CIP | CFF | CAZ | FFC | STR | ENR | TET | TIG | SXT |
| TP22 | Swine faeces | 1. *cibarius* | >128 | ≤0.25 | ≤0.25 | 128 | ≤0.25 | >128 | 128 | 0.5 | 32 | 8 | >4/76 |
| CZP17 | Laying hens feces | *P. mirabilis* | >128 | ≤0.25 | 64 | >128 | ≤0.25 | >128 | >128 | 32 | >128 | 16 | >4/76 |
| CZP26 | Laying hens feces | *P. mirabilis* | >128 | ≤0.25 | ≤0.25 | ≤0.25 | ≤0.25 | >128 | >128 | 1 | 128 | 8 | >4/76 |
| CZP44 | Laying hens feces | *P. mirabilis* | >128 | ≤0.25 | 32 | 32 | 8 | >128 | >128 | 16 | 64 | 8 | >4/76 |
| NYP73 | Laying hens feces | *P. mirabilis* | >128 | ≤0.25 | 4 | ≤0.25 | ≤0.25 | >128 | 128 | 32 | 128 | 16 | >4/76 |
| NYP69 | Laying hens feces | *P. mirabilis* | >128 | ≤0.25 | 4 | ≤0.25 | ≤0.25 | >128 | >128 | 32 | 128 | 16 | >4/76 |
| NYP68 | Laying hens feces | *P. mirabilis* | >128 | ≤0.25 | 16 | 8 | ≤0.25 | >128 | 128 | 32 | 128 | 16 | >4/76 |
| NYP6 | Laying hens feces | *P. mirabilis* | >128 | ≤0.25 | 32 | 8 | ≤0.25 | >128 | 128 | 32 | 128 | 16 | >4/76 |
| ATCC25922 |  | *E. coli* | ≤0.25 | ≤0.25 | ≤0.25 | 0.5 | ≤0.25 | 2 | 2 | ≤0.25 | ≤0.25 | ≤0.25 | <2/38 |

**Abbreviations:** AMX, amoxicillin; MEM, meropenem; CIP, ciprofloxacin; CFF, ceftiofur; CAZ: ceftazidime; FFC, florfenicol; STR, streptomycin; ENR, enrofloxacin; TET, tetracycline; TIG, tigecycline; SXT trimethoprim-sulfomethoxazole.


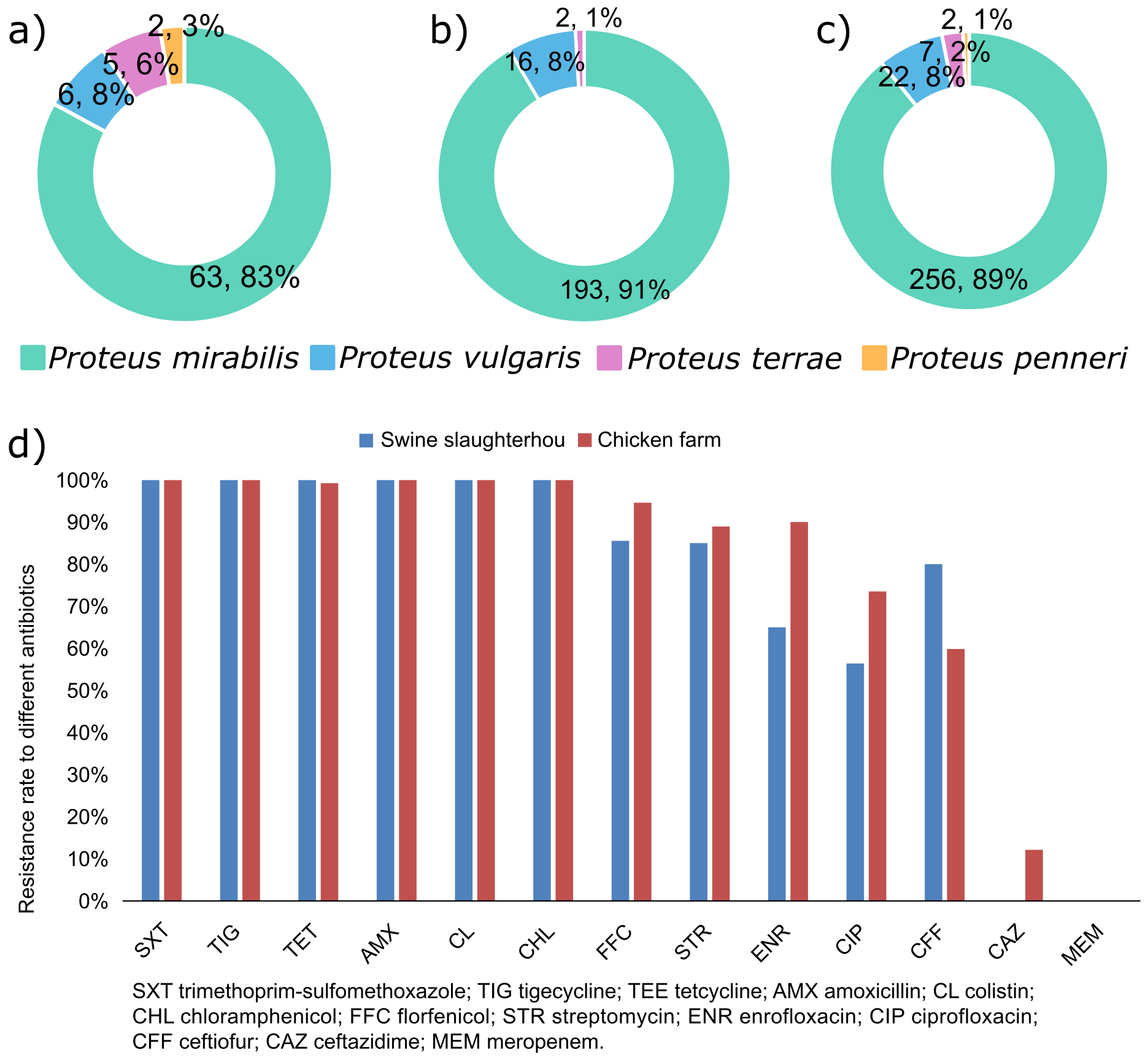


**Figure S1. The bacterial species distribution of 287 ICEs positive *Proteus* spp. and resistance analysis of these isolates.** a) The species composition of 76 *Proteus* spp. from swine slaughterhouse. b) The species composition of 211 *Proteus* spp. from chicken farms. c) The species composition of all of 287 *Proteus* spp. d) The resistance rate to different antibiotics of the 287 *Proteus* spp.


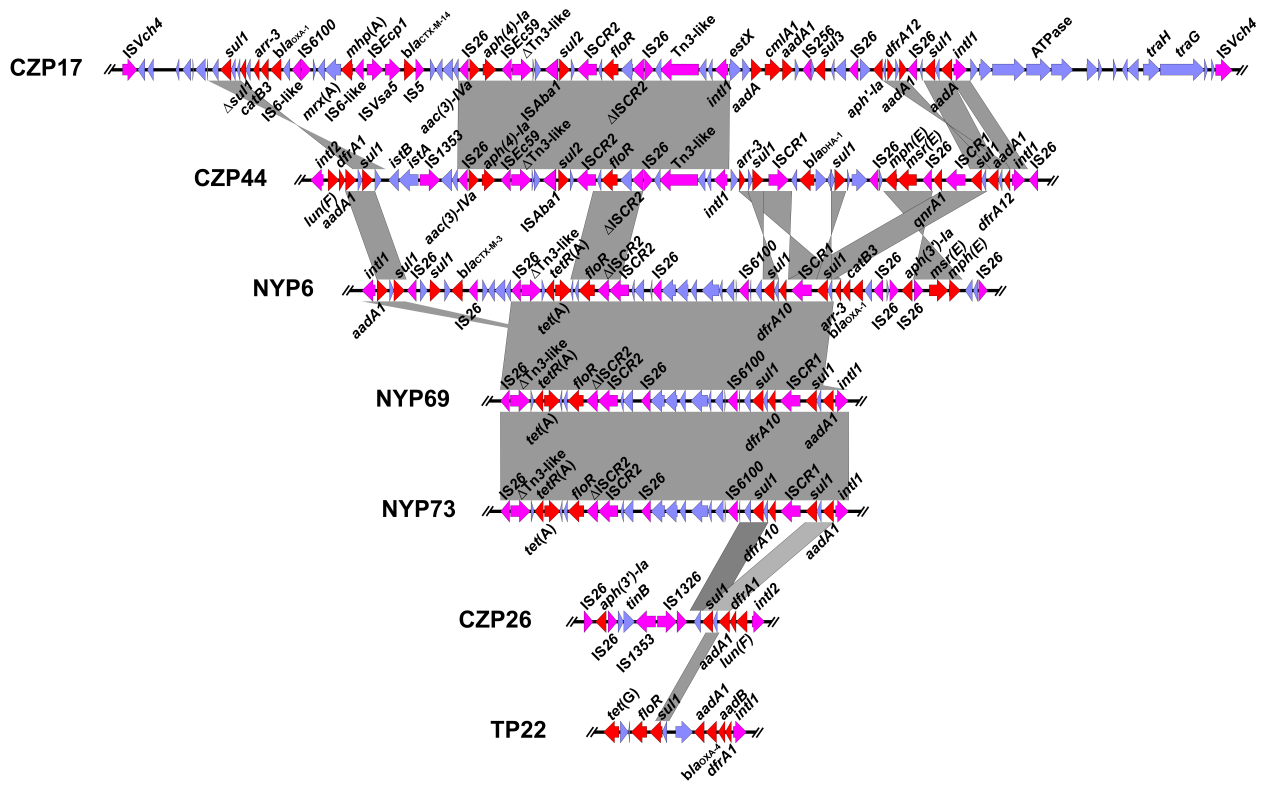


**Figure S2. The** **genetic islands found in chromosomes of *tmexCD3-toprJ1b* positive** ***Proteus* spp.** Majority of genetic islands in chromosomes of *Proteus* spp. were associated with integrons.


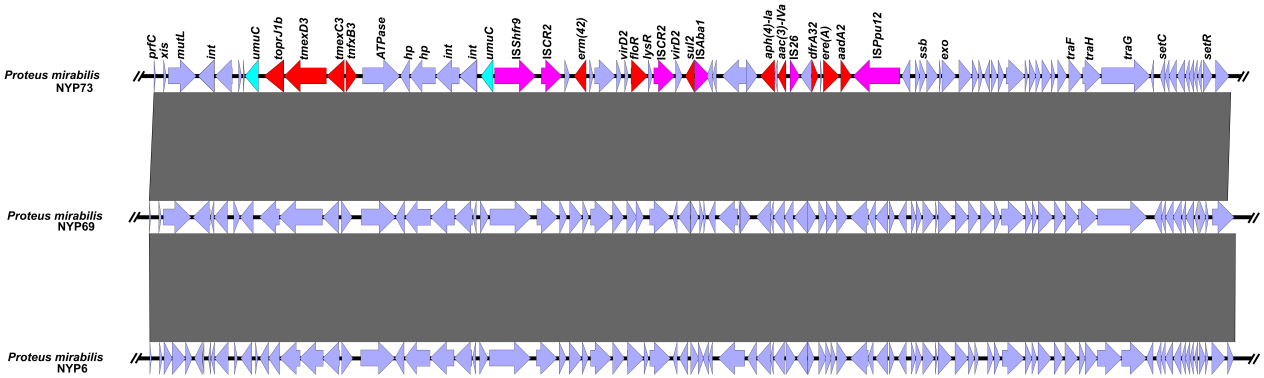


**Figure S3. The genetic structures analysis of ICEs in *tmexCD3-toprJ1b*-bearing isolates from chicken farm of Nanyang, Henan Province.** Red arrows indicate ARGs. The *prfC* gene represents the insertion site of ICEs. The degree of shading indicates the similarity of the four ICEs.


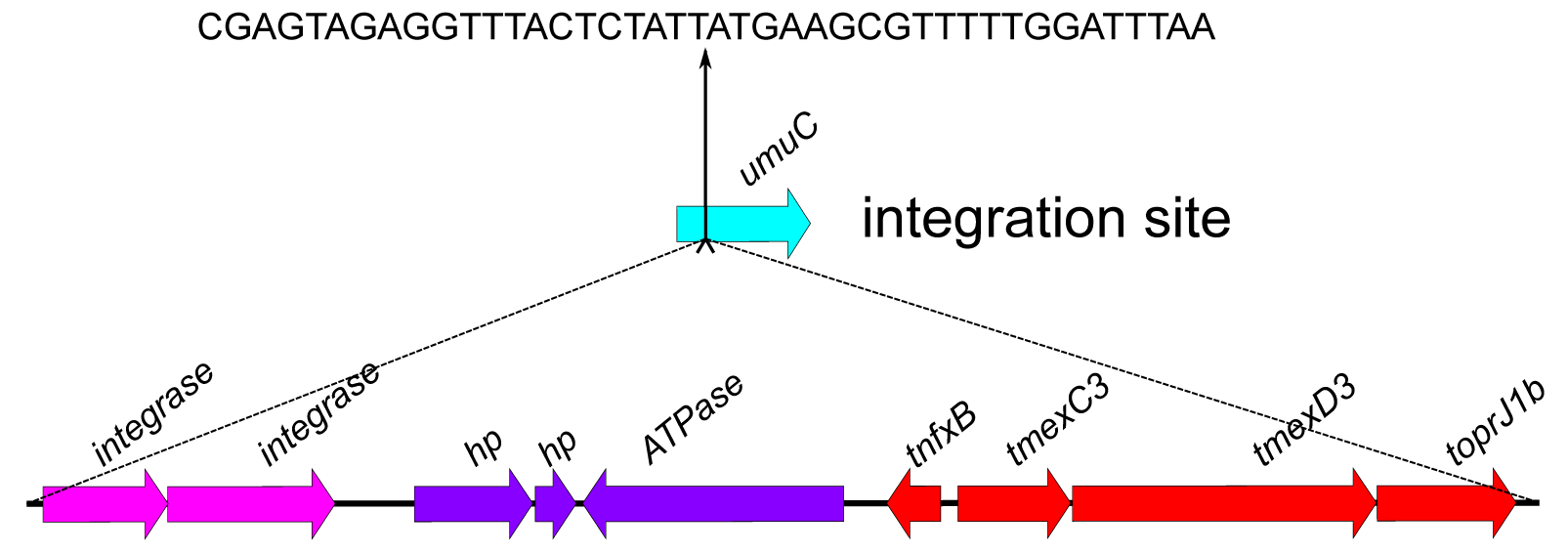


**Figure S4. The conserved integration site of *tmexCD3-toprJ1b* in ICEs of *Proteus* spp.** The *tmexCD3-toprJ1b* gene cluster in all *tmexCD3-toprJ1b* positive *Proteus* spp. was found to be integrated into *umuC* gene of VRⅢ of ICEs. The integration site of *tmexCD3-toprJ1b-*bearing genetic structures in *umuC* gene was highly conserved.
